# Individual differences predict low prevalence visual search performance

**DOI:** 10.1186/s41235-016-0042-3

**Published:** 2017-01-30

**Authors:** Chad Peltier, Mark W. Becker

**Affiliations:** Department of Psychology, Michigan State University, Psychology Building, 316 Physics Rm 298C, East Lansing, MI 48824 USA

**Keywords:** Visual search, Low prevalence, Individual differences

## Abstract

Critical real-world visual search tasks such as radiology and baggage screening rely on the detection of rare targets. When targets are rare, observers search for a relatively short amount of time and have a high miss rate, a pattern of results known as the low prevalence effect. Attempts to improve the search for rare targets have been unsuccessful or resulted in an increase in detections at the price of more false alarms. As an alternative to improving visual search performance through experimental manipulations, an individual differences approach found that those with higher working memory capacity were better at finding rare targets. We build on the individual differences approach and assess 141 observers’ visual working memory capacity (vWMC), vigilance, attentional control, big five personality traits, and performance in both high and low prevalence search tasks. vWMC, vigilance, attentional control, high prevalence visual search performance, and level of introversion were all significant predictors of low prevalence search accuracy, and together account for more than 50% of the variance in search performance. With the exception of vigilance, these factors are also significant predictors of reaction time; better performance was associated with longer reaction times, suggesting these factors identify observers who maintain relatively high quitting thresholds, even with low target prevalence. Our results suggest that a quick and easy-to-administer battery of tasks can identify observers who are likely to perform well in low prevalence search tasks, and these predictor variables are associated with higher quitting thresholds, leading to higher accuracy.

## Significance

Experts who perform important real-world search tasks (e.g., baggage and radiological screening) have been shown to have high miss rates (Fishel, Levine, & Date, 2015). These high miss rates may result from the low prevalence effect—the finding that miss rates increase dramatically when targets are rare. Attempts to alter search procedures to minimize the low prevalence effect have had limited success (Wolfe et al., 2007). However, there is some indication that individual differences in working memory capacity (WMC) can predict performance in low prevalence search tasks (Schwark, Sandry, & Dolgov, 2013). Here we expand on this work to identify a battery of tasks that are predictive of an individual’s performance on a low prevalence search task. To maximize utility in an applied setting, the tasks we chose to evaluate were easy to administer via computer and were fairly quick to complete. The results of our regression analysis suggest that five factors (measures of visual WMC, attentional control, vigilance, high prevalence search performance, and the personality trait of introversion) were significant predictors of low prevalence search performance; a regression model with these five factors accounted for over 50% of the variance in low prevalence visual search performance. Critically, these tasks predicted an increase in the hit rate, without an associated increase in false alarms, showing a beneficial shift in sensitivity without a detrimental shift in the criterion. We propose that these tasks could be used by employers to identify individuals who are suitable to perform critical, real-world low prevalence searches.

## Background

Rare targets are missed often in the laboratory and in the real world. When untrained observers search for rare targets (at 10% prevalence or below), miss rates can reach or exceed 40% (Peltier & Becker, 2016; Rich et al., 2008; Wolfe, Horowitz, & Kenner, 2005). This high miss rate in the laboratory mirrors the difficulties that radiologists and baggage screeners face; they have miss rates as high as 30% and 95%, respectively (Evans, Birdwell, & Wolfe, 2013; Fishel et al., 2015), while searching for targets with prevalence rates as low as 0.3% (Gur et al., 2004).

Given the high costs of misses in real-world search scenarios like baggage screening and radiology, several attempts have been made to improve the search for rare targets. Wolfe et al. (2007) performed seven experimental manipulations in an attempt to alleviate the low prevalence effect. These manipulations included having two searchers who worked together on the same visual search task, forcing slower responses, introducing high prevalence targets to boost detection of low prevalence targets, searching for multiple categories with different prevalence rates, having trials with multiple targets, and having periods of higher target prevalence. The only manipulation which increased rare target detection was introducing bursts of high prevalence trials with feedback among the low prevalence blocks without feedback. However, this method also increased false alarms, which can be costly errors in both radiology and baggage screening. In addition, this pattern suggests that the manipulation did not change sensitivity, but only shifted the decision criterion.

In a similar manipulation, Schwark, Sandry, Macdonald, and Dolgov (2012) attempted to increase rare target detection rates by providing misleading feedback that increased the perceived prevalence of targets. The researchers increased one group of observers’ perceived prevalence rates by informing them they had missed the target on 20% of correctly rejected trials. Similar to Wolfe et al. (2007), this manipulation increased the hit rate, but also increased the rate of potentially costly false alarms, suggesting that it produced a shift in decision criterion rather than an increase in sensitivity.

Kunar, Rich, and Wolfe (2010) also attempted to increase the rare target detection by presenting half of the items in the display at one time, then adding the remaining half after 1000 ms. In different experiments the two halves were separated spatially, or presented spatially intermixed. Both methods failed to increase rare target detection.

Fleck and Mitroff (2007) proposed that the low prevalence effect is due to observers executing the prepotent “target-absent” motor response despite detecting the target, and thus offered observers a corrective response in the case of an accidental button press. Although they found that the corrective response eliminated the low prevalence effect, this result has not been replicated by other researchers (Peltier & Becker, 2016; Van Wert, Horowitz, & Wolfe, 2009) and one of the original researchers later published a paper that reported the low prevalence effect (Mitroff & Biggs, 2014). Thus it appears that allowing a corrective response does not eliminate the low prevalence effect.

The purpose of listing these failed attempts to minimize the low prevalence effect is to illustrate the variety of methods that have been attempted and highlight how difficult it has been for researchers to improve the search for rare targets. Given the difficulty of improving an individual’s search performance, an alternative approach is to identify those individuals who are better at low prevalence search tasks. If a screener could identify individuals who were likely to be particularly good at detecting rare targets, employers could screen for those employees, thereby improving overall target detections. For such a screener to be effective, one would need to show that there are sizeable individual differences in rare search target detection rates, and one would need to find individual difference variables that were predictive of rare target detection rates.

Research by Schwark et al. (2013) provides data suggesting that both of these requirements might be met. Schwark et al. (2013) found that there are large individual differences in low prevalence search performance, with hit rates ranging from 0% (3 subjects out of 40) to 100% (8 subjects). Given these large individual differences in performance, they investigated whether individual differences in WMC could predict low prevalence search performance. They found a significant relationship between WMC and low prevalence search performance, such that those with high WMC had higher hits rates and slower target-absent search times. They attributed the high target detection rate among those with higher WMC to maintaining high quitting thresholds in low prevalence tasks, meaning those with higher WMC searched longer for a target before terminating the search.

Encouraged by their work, here we attempt to identify additional predictors of rare target detection, in the hopes of developing a screener that can be used to identify people who would be particularly good at detecting rare targets. Given that there has been limited research investigating predictors of low prevalence visual search performance, this work is largely exploratory. We chose a handful of cognitive tasks designed to measure factors that we thought might be associated with rare search accuracy. These include measures of visual working memory capacity (vWMC), vigilance, attentional control, and performance on moderate prevalence search. We also investigated whether any of the big five personality factors could add additional ability to identify individuals who would be good at low prevalence search. Ultimately, our goal is to assemble a battery of tasks that can be used to reliably predict low prevalence search accuracy.

While our main goal was to find predictors of search accuracy, we also investigated how these factors were related to reaction time and false alarm rates. The reason we did this was to investigate the potential mechanisms by which the predictors influence target detection rates. If the predictors of good target detection are also predictors of slower search reaction times, the data would be consistent with the mechanism being the individual’s quitting threshold; predictors of a high quitting threshold should be associated with both slower reaction times and better target detection. By contrast, if the mechanism was simply a change in the decision criterion for the responding target present, then predictors of higher target detection rates should also be predictive of more false alarms. In short, investigating these two additional dependent variables provides preliminary evidence about the potential underlying mechanism by which the predictors influence low prevalence search rates.

## Methods

### Participants

One hundred and fifty-eight undergraduates (109 females) from Michigan State University’s human subjects pool gave consent to participate in the study for course credit. All subjects were between the ages of 18 and 24 with normal or corrected to normal vision. Fourteen subjects were excluded from further analysis for failing to complete all tasks.

### Low prevalence visual search task

**Fig. 1:**
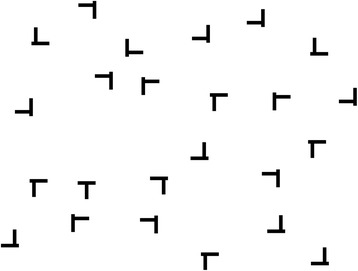
Example image from the visual search task. The target T is in the lower left quadrant

The task was to search for a rotated T among an array of 24 items and respond present or absent via button press (see Fig. 1). Distractors were rotated, offset L symbols. The use of these offset L symbols makes the search task far more difficult and less efficient than the typical T among L symbols search task. In target-absent trials, all 24 stimuli were distractors. In target-present trials, one randomly chosen L was replaced with a rotated T. The orientation of each item was randomly assigned to be 0, 90, 180, or 270° from vertical.

Each item subtended 1.2° × 1.2° of visual angle. To create each array, the screen was divided into 24 (6 × 4 matrix) equal-sized (6.4° × 7.1°) regions. A single item was placed within each region, with random jitter that allowed the item to appear anywhere within the region. This jitter broke up the orderly organization of the matrix and resulted in the items appearing in different locations across trials.

In this low prevalence task there were 27 target-present trials randomly interleaved with 243 target-absent trials for a prevalence rate of 10%. The block of trials was preceded by 50 practice trials with a 10% target prevalence rate in order to allow search parameters (quitting threshold and decision criterion) to be set for a low prevalence task (Ishibashi, Kita, & Wolfe, 2012; Wolfe & Van Wert, 2010).

### Cognitive predictor tasks

#### High prevalence visual search task

Finding that high prevalence search performance is a good predictor of low prevalence search performance would provide an efficient method for predicting who would be good at low prevalence search tasks. For instance, the target prevalence rate in cancer screening is ~0.3% (Gur et al., 2004). Thus it would take several thousand trials to gather reliable data about an observer’s performance at these low prevalence levels. However, if high prevalence search performance is a good predictor of low prevalence search performance, then one could gather data about an individual’s performance very quickly at a high prevalence rate and use that as a predictor of the unobserved low prevalence performance. The inclusion of this high prevalence block of trials also allowed us to confirm that our subjects were demonstrating the traditional low prevalence effect (Wolfe et al., 2005).

The high prevalence visual search task was identical to the low prevalence task, except that the target prevalence rate was set at 50%. Like the low prevalence task, there were 27 target-present trails, for a total of 54 trials in the block. The block of trials was preceded by 50 practice trials with 50% prevalence rate in order to allow search parameters (quitting threshold and decision criterion) to be set for a 50% prevalence task (Ishibashi et al., 2012; Wolfe & Van Wert, 2010). To calculate high prevalence performance, the predictor variable we use in the analyses, we subtracted false alarms from hits for each participant.

#### Working memory capacity

Schwark et al. (2013) found a positive relationship between performance on the AOSPAN task (Unsworth, Heitz, Schrock, & Engle, 2005) and low prevalence hit rate. They interpreted this as evidence that vWMC was related to rare target detection. However, the AOSPAN task most likely measures both capacity and executive attention (Unsworth et al., 2005). To obtain a more pure measure of capacity, we used the change detection task popularized by Vogel, Woodman, & Luck (2001) to measure vWMC.

Observers viewed a display of four, six, or eight colored squares for 100 ms and tried to remember the color and location information during a 900 ms retention interval. After the retention interval, a single-colored probe square appeared in one of the previously occupied locations. Participants had to indicate whether the color of the probe square matched the color at that location during the original display. The task consisted of 120 trials and took approximately 10 minutes to complete. We used the formula from Pashler (1988) to calculate each subject’s capacity (*K*) from the participant’s accuracy data.

#### Vigilance

Vigilance is the ability to sustain attention over long periods of time (Chun, Golomb, & Turk-Browne, 2011). Given that low prevalence search tasks require observers to maintain the attentional goal of detecting the target over long periods of time, a task that can quickly assess vigilance may be a useful and valid predictor of low prevalence search.

We measured vigilance using a go/no-go continuous performance task (Covey, Shucard, Violanti, Lee, & Shucard, 2013). A total of 410 letters were presented one at a time at fixation for 400 ms each with a 1500 ms interval between letters, for a total task duration of approximately 15 minutes. Observers were instructed to make a button press when they detected an X that was preceded by an A (go trial), which happened 40 times across the sequence of 410 letters. The letters A and X appeared an additional 40 times each without being paired together in the A then X “go” combination. Each observer’s vigilance score was calculated by subtracting the corrected hit rate (hits minus false alarms) in the first quarter of trials from the last quarter of trials.

#### Posner cuing

Serial visual search tasks, such as the task we use, require a series of endogenous attentional shifts from item to item (Wolfe, 1994). This process involves disengagement from the currently attended item, a shift in spatial attention, and then reengagement onto the new item. Those who are fast to perform these processes may be faster or more effective in visual search tasks.

We used a modified Posner cuing task (Posner, 1980) with a central endogenous cue to measure reaction to validly cued, neutral, and invalidly cued targets. The trial sequence consisted of a fixation point, followed by a central arrow cue that pointed to the left or the right, followed by a black square that could appear on the left or right of fixation. Observers were to report the location of the target (left/right) as quickly and accurately as possible. In half of the trials the arrow cue was a neutral cue consisting of a two-headed arrow that pointed to both potential target locations. In the remaining half of the trials, the cue was a unidirectional arrow that pointed to the eventual target location (valid cue) 75% of the time. The cue pointed to the wrong location (invalid cue) in the remaining 25% of unidirectional cue trials.

Cues appeared for 250 ms and targets appeared for 100 ms with a 550 ms blank between cue and target. The time between target presentation and the start of the next trial randomly varied between 3000, 4000, and 5000 ms. Observers responded whether the target was on the right or left side using a button press. The task consisted of 108 trials and took approximately 15 minutes to complete. In later text, this variable will be referred to as attentional control, and was calculated using the difference between invalid and valid trials’ reaction times in correct trials.

### Personality predictors

Personality traits are a potential predictor of visual search performance. There is conflicting evidence over whether introverts are better at searching (Sen & Goel, 1981) or perform equally well in comparison with extraverts (Newton, Slade, Butler, & Murphy, 1992). However, a meta-analysis of 53 studies (Koelega, 1992) has shown that introverts consistently perform better than extraverts on tasks requiring sustained attention as measured by the hit rate. Introverts also show a smaller performance decrement as time on task increases. These results can potentially be attributed to the theory that introverts have a higher base level of arousal, which allows them to perform monotonous tasks (like low prevalence searches) at a high level (Eysenck, 1967).

Other personality factors may be of interest as well. A meta-analysis of 65 studies (Judge & Ilies, 2002) investigated the relationship between the Big Five personality traits and performance motivation. The study showed that neuroticism and conscientiousness were the best predictors of performance motivation. It is possible that performance motivation predicts effort and quitting thresholds in low prevalence searches and thus could be correlated with accuracy. In sum, personality factors are reasonable candidate predictors of visual search performance and thus were included in our design.

In accordance with our effort to build a battery of measurements of maximum utility in a real-world setting where speed of assessment is important, we measure the Big Five personality traits using the Mini IPIP (Donnellan, Oswald, Baird, & Lucas, 2006), which has only 20 questions. The Mini IPIP is similar in reliability, convergent validity, and criterion validity measures to longer personality assessments, while still tapping almost the same content, despite being an abbreviated assessment (Donnellan et al., 2006).

### Procedure

Observers first completed the two blocks (high prevalence and low prevalence) of visual search trials. The order of these blocks was randomized for each subject. Observers then completed the following tasks in the same order: change detection, vigilance, Posner cuing, and MINI IPIP.

All tasks were programmed in E-prime, and presented individually in sound attenuated booths, on PCs with 20-inch CRT monitors set at a resolution of 1024 × 768 with a 100 Hz refresh rate. Each task began with onscreen instructions about the upcoming task, and participants were able to take brief breaks between each task.

## Results

Prior to conducting the following analyses, we filtered our data for outliers. This included eliminating subjects who had studentized deleted residual values >3 or Cook’s distance values >1 (Cook & Weisberg, 1982). These subjects are considered outliers or points that have high leverage on the model fit. This eliminated three subjects from further analyses, leaving us with a final sample size of 141. Visual search trials with a reaction time beyond three standard deviations from the mean for each subject at each prevalence rate were also discarded from further analysis, resulting in the exclusion of 1.1% of low prevalence trials and 0.6% of high prevalence trials. Means and zero-order correlations for all variables are summarized in Table 1.

**Table 1:** Means (SEMs) and zero-order correlations of and between all variables

	LowPrev ACC	LowPrev AbsentRT	HighPrev ACC	vWMC (*K*)	Vigilance	Attn Control	I	C	E	A	N
LowPrev ACC	1	0.79*	0.67*	0.29*	0.29*	0.28*	–0.19*	–0.04	–0.12	–0.13	–0.02
LowPrev AbsentRT	0.79*	1	0.56*	0.25*	0.25*	0.225*	–0.08	–0.1	–0.12	–0.07	–0.04
HighPrev ACC	0.67*	0.56*	1	0.18*	0.21*	0.11	–0.09	–0.04	0.04	–0.04	0.08
vWMC (*K*)	0.29*	0.25*	0.18*	1	0.11	0.21*	–0.09	–0.07	0.005	0.01	0.07
Vigilance	0.29*	0.25*	0.21*	0.11	1	0.20*	0.05	–0.09	–0.08	0.02	–0.05
Attn Control	0.28*	0.225*	0.11	0.21*	0.20*	1	–0.18*	–0.02	0.02	0.24*	–0.04
I	–0.19*	–0.08	–0.09	–0.09	0.05	–0.18*	1	0	0.133	0.03	0.01
C	–0.04	–0.1	–0.04	–0.07	–0.09	–0.02	0	1	0.03	0.10	–0.12
E	–0.12	–0.12	0.04	0.005	–0.08	0.02	0.133	0.03	1	0.38*	0.08
A	–0.13	–0.07	–0.04	0.01	0.02	0.24*	0.03	0.10	0.38*	1	–0.06
N	–0.02	–0.04	0.08	0.07	–0.05	–0.04	0.01	–0.12	0.08	–0.06	1
Mean (SEM)	0.40 (0.018)	4879.58 ms (68.50)	0.56 (0.018)	1.89 (0.078)	0.023 (0.008)	60.2 ms (4.71)	3.49 (0.04)	3.66 (0.07)	3.18 (0.08)	4.13 (0.05)	2.93 (0.05)

Attentional control and vigilance measures are from the subtractions described in the respective cognitive predictors sections

*p < 0.05

*LowPrevACC* hits minus false alarms in the low prevalence condition, *LowPrev AbsentRT* reaction time in target-absent trials in the low prevalence condition, *HighPrevACC* hits minus false alarms in the high prevalence condition, *vWMC (*K*)* visual working memory capacity represented as *K*, *Vigilance* vigilance score as assessed by the vigilance task, *Attn Control* attentional control as assessed by the Posner Cuing task, *I* intelligence/openness to experience, *C* conscientiousness, *E* extraversion, *A* agreeableness, *N* neuroticism

### The low prevalence effect: reaction time and accuracy

To verify that we observed the traditional low prevalence effect, we analyzed the data from the visual search blocks using two 2 (target present/target absent) × 2 (low/high prevalence block) repeated-measures ANOVAs, one on the corrected hit rate (percentage hits minus percentage false alarms) data and one on the reaction time data. The ANOVAs were followed-up with planned paired-sample *t* tests to verify the presence of the low prevalence effect.

**Fig. 2:**
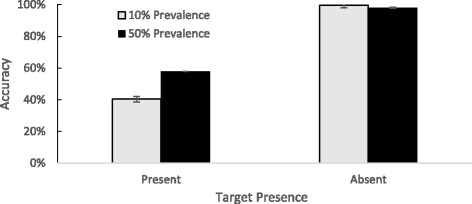
Accuracy by target prevalence and presence. *Error bars* represent the standard errors of the means

We found standard prevalence effects on accuracy (see Fig. 2); as target prevalence decreased, the proportion of misses increased. This was confirmed by a significant interaction between target prevalence and target presence, *F*(1, 140) = 165.87, *p* < 0.001, η_p_^2^ = 0.54, such that hit rate was lower in low prevalence trials. Pairwise comparisons show that the 10% prevalence hit rate (M 0.40, SEM 0.018) was significantly lower than the 50% prevalence hit rate (M 0.58, SEM 0.016), *t*(140) = 12.31, *p* < 0.001, *d* = 2.08. Pairwise comparisons also show higher correct rejection rate in the 10% prevalence trials (M 0.996, SEM 0) than in the 50% prevalence trials (M 0.98, SEM 0.004), *t*(140) = 4.73, *p* < 0.001, *d* = 0.80. Although the difference between the correct rejection rate at the two prevalence rates was significant, there was a larger effect of prevalence on hit rate.

**Fig. 3:**
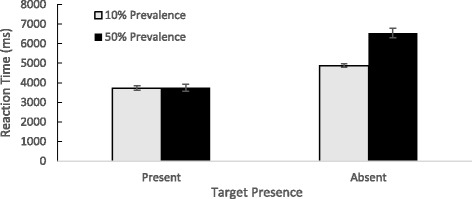
Reaction time by target prevalence and presence. *Error bars* represent the standard errors of the means

Similar to previous results, the reaction time data found that target prevalence had its primary effect on target-absent trials (Ishibashi et al., 2012; Rich et al., 2008); as prevalence decreased, reaction times in target-absent trials also decreased (see Fig. 3). This was confirmed by a significant interaction between target prevalence and target presence, *F*(1, 140) = 103.23, *p* < 0.001, η_p_^2^ = 0.42, driven by a much larger prevalence effect in target-absent trials. Pairwise comparisons show that target-absent reaction times in the 10% prevalence block (M = 4879.58, SEM = 168.50) were significantly faster than in the 50% prevalence block (M = 6540.76, SEM = 239.18), *t*(140) = 7.80, *p* < 0.001, *d* = 1.32. A pairwise comparison of target-present trials showed no difference between 10% (M = 3725.84, SEM = 109.11) and 50% (M = 3749.21, SEM = 94.65), *t*(140) = –0.22, *p* = 0.83, *d* = 0.037, confirming that the interaction was caused by a drop in target-absent reaction time as prevalence decreased.

### Predicting accuracy by cognitive factors

To investigate the ability of individual difference factors to predict low prevalence search performance, we performed linear regression models. For all regression models, we ensured that the assumptions of the regression models were met in the following manner. Linearity was assessed through visual inspection of partial regression plots and a plot of studentized residuals against the predicted values. Homoscedasticity was assessed by visual inspection of a plot of studentized residuals versus unstandardized predicted values. Normality was assessed by visual inspection of the P–P plot. Durbin–Watson statistics were all between 2.05 and 2.19, while tolerance values ranged from 0.77 to 0.93, thus showing no violations of independence of residuals or multicollinearity.

We then used our cognitive measures as predictors in a multiple regression model to predict low prevalence accuracy. The cognitive predictors that were entered into this model included high prevalence search performance, *K*, vigilance, and attentional control. The multiple regression model significantly predicted low prevalence accuracy, *F*(4, 136) = 36.29, *p* < 0.001, adjusted *R* ^2^ = 0.502. High prevalence performance, *K*, and attentional control all significantly contributed to the model, *t* values > 2.2, *p* values < 0.03, all β values > 0.11. Vigilance marginally contributed to the model, *t* = 1.823, *p* = 0.07, β = 0.11, and was included in the overall model.

### Accuracy regression: personality factors

After validating our cognitive predictors of accuracy, we enter in the personality factors as measured by the Mini IPIP (Donnellan et al., 2006) into the second stage of a hierarchical linear regression (see Appendix Table 5 for the full model). We separate the entry of personality and cognitive predictors into the regression model to show the benefit of including personality measures in predicting visual search performance. The new multiple regression model, *F*(9, 131) = 17.94, *p* < 0.001, adjusted *R* ^2^ = 0.521, marginally predicted low prevalence accuracy over and above the cognitive factors regression model, adjusted *R* ^2^ change = 0.019, *F* change = 2.098, significant *F* change = 0.07. Extraversion was a marginally significant predictor of rare target detection accuracy, *t* = –1.97, *p* = 0.051, β = –0.13, with those higher rates of introversion predicting better target detection. All other personality measures did not approach being significant predictors, all *t* values < 1.43, all *p* values > 0.15. For our final model we thus reduced the model to included only the cognitive factors and the extraversion personality factor.

### Overall accuracy regression model

A multiple regression model was used to low prevalence search accuracy^1^ from the factors high prevalence search performance, *K*, vigilance, attentional control, and extraversion. The overall accuracy regression model predicted low prevalence accuracy, *F*(5, 135) = 31.68, *p* < 0.001, adjusted *R* ^2^ = 0.523. All factors significantly contributed to the model (see Table 2).

**Table 2:** Low prevalence accuracy regression results

	*t*	*p* value	β
High prevalence performance	9.99	<0.001	0.60
*K*	2.27	0.03	0.14
Vigilance	2.05	0.04	0.13
Attentional control	2.59	0.03	0.14
Extraversion	–2.63	0.009	–0.15

*t*, *p*, and β values for each predictor in the final regression model for low prevalence accuracy

### Reaction time regression: cognitive factors

A multiple regression model was used to predict the low prevalence reaction time from the factors high prevalence search performance, *K*, vigilance, and attentional control. The multiple regression model significantly predicted the low prevalence target-absent reaction time, *F*(4, 136) = 19.97, *p* < 0.001, adjusted *R* ^2^ = 0.351.

High prevalence performance was a significant predictor of low prevalence target-absent reaction time, *t* = 7.16, *p* < 0.001, β = 0.51. *K* and attentional control were marginally significant predictors of low prevalence reaction time, both *t* values > 1.74, both *p* values < 0.084, β values > 0.12. Vigilance was a nonsignificant predictor, *t* = 1.48, *p* = 0.14, β = 0.10. We enter the cognitive factors high prevalence performance, *K*, and attentional control into the model with personality factors.

### Reaction time regression: personality factors

Personality factors were entered into the second stage of a hierarchical linear regression after the cognitive factors (see Appendix Table 6 for full model). The new multiple regression model, *F*(8, 132) = 10.85, *p* < 0.001, adjusted *R* ^2^ = 0.36, did not predict low prevalence accuracy over and above the cognitive factors regression model, adjusted *R* ^2^ change = 0.014, *F* change = 1.62, significant *F* change = 0.16.

Of the personality factors, only extraversion marginally predicted low prevalence target reaction time, *t* = –1.94, *p* = 0.055, β = –0.15, such that introverts have greater reaction time, and thus is the only personality factor included in our final regression model.

### Overall reaction time regression model

A multiple regression model was used to predict low prevalence reaction from the factors high prevalence performance, *K*, attentional control, and extraversion. The hierarchical reaction time regression model predicted the low prevalence target-absent reaction time, *F*(4, 136) = 20.7, *p* < 0.001, adjusted *R* ^2^ = 0.36, over and above the cognitive model alone, adjusted *R* ^2^ change = 0.014, *F* change = 4.09, significant *F* change = 0.045. High prevalence performance, attentional control, and extraversion significantly contributed to the model and *K* was a marginal predictor (see Table 3).

**Table 3:** Low prevalence reaction time regression results

	*t*	*p* value	β
High prevalence performance	7.68	<0.001	0.53
*K*	1.89	0.06	0.13
Attentional control	2.01	0.04	0.14
Extraversion	–2.02	0.04	–0.14

*t*, *p*, and β values for each predictor in the final regression model for low prevalence target-absent reaction time

### False alarms

The goal of this research was to find those who have high accuracy, which is made up of both a high hit rate and a low false alarm rate. Because of our measure of low prevalence accuracy (hits minus false alarms), it is possible that observers rated high on the measures high prevalence performance, *K*, attentional control, vigilance, and introversion simply increased their hits more than their increase in false alarms, which could increase accuracy as a result of a shift in the criterion. However, false alarms in cancer screening and security are costly, thus it would be ideal if our measures predicted an increase in hits without a corresponding increase in false alarms, which was the imperfect experimental solution to the low prevalence effect (Wolfe et al., 2007). To test this possibility, we correlated each significant predictor of low prevalence accuracy with false alarms. We found high prevalence performance and *K* are both significantly correlated with false alarms (see Table 4). Importantly, the direction of that relationship is such that those who have higher scores on these measures make fewer false alarms, suggesting that they have higher sensitivity, rather than a liberal criterion.

**Table 4:** Correlations between significant accuracy predictor variables and false alarms

	High prevalence performance	Attentional control	*K*	Vigilance	Extraversion
Pearson correlation	–0.321	0.149	–0.174	0.05	0.095
*p* value	<0.001	0.082	0.039	0.05	0.261

Correlations and *p* values for the relationships between significant predictors of low prevalence accuracy and low prevalence false alarms

### Predictors of high prevalence accuracy

In finding predictors of low prevalence search performance, it is possible that we did not identify tasks which predict who is uniquely suited for performing critical low prevalence search tasks, but perhaps found those who are generally good at searching. If this alternative interpretation of our results is true, we should find that each variable which accounts for a significant proportion of low prevalence performance should also account for a significant proportion of high prevalence performance.

To test for this possibility, we performed a linear regression predicting high prevalence search performance using low prevalence search performance, *K*, vigilance, attentional control, and extraversion.^2^ We found that of these predictors, only low prevalence search performance, *t* = 9.99, *p* < 0.001, β = 0.70, accounted for a significant portion of high prevalence performance, while extraversion was a nearly significant predictor, *t* = 1.9, *p* = 0.06, β = 0.12. All other predictors were not significant, *t* values < 1.3, *p* values > 0.19, β values < 0.08. From these data we conclude that our predictors of low prevalence performance are uniquely suited to account for low prevalence search performance, not visual search in general.

## Discussion

Using an individual differences approach, we found that better accuracy in a low (10%) prevalence search task was predicted by higher accuracy on a high (50%) prevalence search task, higher vWMC, more vigilance, and more rapid attentional shifting. When we then added personality measures to the regression model, only the extraversion factor increased the model’s predictive ability, with more introverted participants tending to perform better on the low prevalence search task. A final regression model with these five factors accounted for more than half of the variance in rare target search performance.

Each of our predictors were chosen because of their relation to visual search. vWMC has been shown to predict quitting thresholds and hit rate in low target prevalence (Schwark et al., 2013). Vigilance tasks and low target prevalence search share the need to maintain attention while trying to detect the rare target. Similarly, research has claimed that introverts maintain a higher level of baseline arousal (Eysenck, 1967), which allows them to perform monotonous tasks, such as a low prevalence search task, at a high level for prolonged periods. Visual search and our measure of attentional control both require the shifting of attention between stimuli. Although we identified some valid predictors of low prevalence search here, later efforts should seek to expand on this battery with additional predictor tasks.

Importantly, both increased vWMC and higher accuracy in the high prevalence search performance predicted fewer false alarms in the low prevalence search task, and the remaining predictor variables were not significant predictors of false alarms. This finding that the predictors associated with better low prevalence target detection were also associated with fewer false alarms suggests that these factors predict an increase in sensitivity, rather than only a shift in the decision criterion for responding “target present”.

In addition, we found that each of the factors that predicted better target detection, with the exception of vigilance, also predicted slower low prevalence target-absent reaction times. This pattern of results hints at a potential underlying mechanism that mediates the relationship between our predictors and rare target search performance, namely that the predictors may be systematically related to an individual’s quitting threshold. Theoretical models designed to explain how target-absent responses occur in visual searching propose that, during the course of a trial, evidence accumulates toward a trial quitting threshold (Wolfe & Van Wert, 2010). If this accumulation of evidence reaches the quitting threshold prior to the identification of a target, a target-absent response is made (Wolfe & Van Wert, 2010). In theory, as targets become rare the quitting threshold decreases, resulting in the need to examine less of the display before executing a target-absent response, and leading to increased miss errors (Hout, Walenchok, Goldinger, & Wolfe, 2015). In short, a lower quitting threshold is associated with both lower target-absent reaction times and worse target detection accuracy. The fact that our predictors are associated with both of these outcomes provides a hint that the predictors may be related to an individual’s low prevalence quitting threshold, providing potential insight into the mechanism by which these predictors mediate low target search performance.

It is worth noting that the most powerful predictor of low prevalence search accuracy was high prevalence search performance. While this may not be surprising because these tasks represent near transfer, it has important practical implications. Real-world searches can have target prevalence rates as low as 0.3%, thus using a realistic target prevalence rate in a screener task would be time consuming and expensive because potential employees would have to complete several thousand trials to gather reliable data about their performance. Given the strong association between high and low target prevalence accuracy, using one’s performance on a high prevalence search task as a proxy for their likely ability to detect low prevalence targets may be a much more economical approach. However, it is worth pointing out that the other factors, leaving out high prevalence search performance, constitute a significant model on their own. When removing the high prevalence search factor from the regression model, the cognitive factors are all still significant predictors (all *t* values > 2.28, all *p* values < 0.025), and extraversion is a marginally significant predictor (*t* = –1.83, *p* = 0.07) of low prevalence search accuracy.

The current work demonstrates that approaching the problems associated with low target prevalence from an individual differences perspective has promise. These predictor tasks may have real-world significance in that they can be used to find those who are more suited for tasks where the goal is to find a rare target, such as in airport security checks. Each task is fast and easy to administer, maximizing its potential to be used in the workplace. The finding that these factors all predict higher accuracy without a significant increase in false alarms shows that an individual differences approach may be more suitable to increase accuracy in these situations than the currently known experimental manipulations (Kunar et al., 2010; Wolfe et al., 2007). Given this promise, future investigations pursuing individual differences to identify those who would be good at low prevalence search are warranted.

Among the questions this future research should investigate is the extent to which the prediction model we have identified would generalize to predicting expert performance, searches for critical real-world targets (e.g., cancers in radiological scans and weapons in baggage scans), and more real-world work environments (Clark, Cain, Adamo, & Mitroff, 2012). Our experiment uses 10% target prevalence as the low prevalence condition, but research has shown that the prevalence effect (i.e. increase in miss rates) becomes more pronounced as prevalence decreases further (Wolfe et al., 2005), meaning that we may be underestimating the effects of prevalence. However, our data suggest that as prevalence decreases, the strength of the predictors increases. If this trend continues to even lower prevalence rates, such as the 0.3% prevalence found in breast cancer screening (Gur et al., 2004), then the predictors may be even stronger. Alternatively, we could find that our predictors’ strength is overestimated when predicting expert performance because training may overcome basic individual differences; although in the expertise literature there is evidence suggesting that initial abilities, such as WMC, predict performance over and above practice (Meinz & Hambrick, 2010). Additionally, real-world searches depend on the observer’s ability to find multiple different targets, whereas our observers only need to search for a target T. Future research addressing these concerns would be required to establish the value of implementing these types of screening tools in a real-world context.

Despite the need for future research, our current investigation has both practical and theoretical relevance. In terms of the practical, a screener that uses the five factors of our model to identify people who would be likely to perform well in situations that require the detection of low prevalence targets, like baggage screeners, may significantly increase target detection. In terms of the theoretical, the pattern of relationships between our predictors and both accuracy and target-absent reaction time suggest that our predictors might be associated with an individual’s low prevalence quitting threshold, hinting at a potential mechanism for the relationships.

## Conclusions

Critical real-world search tasks (i.e., radiology and baggage screening) rely on observers to detect rare targets, but observers often fail to do so (Fishel et al., 2015). Our battery of tasks builds on previous individual differences approaches that aim to increase search performance in these important situations. By measuring individual differences in vWMC, attentional control, high prevalence search performance, vigilance, and intraversion, we can predict who is uniquely suited to perform low prevalence searches. These tasks accounted for more than half the variance in low prevalence accuracy, while taking only 1 hour to complete, showing this battery of tasks to be both effective and quick to complete. Critically, these tasks predict an increase in hit rate without an increase in false alarms, which could be costly in an applied setting. Our results suggest that employers could use our methodology to screen for those who are likely to perform low prevalence search tasks effectively.

## Appendix

**Table 5:** Complete low prevalence accuracy regression results

	*t*	*p* value	β
High prevalence performance	9.85	<0.001	0.60
*K*	2.35	0.02	0.14
Vigilance	2.11	0.04	0.13
Attentional control	2.23	0.03	0.14
Intelligence	–1.09	0.27	–0.06
Conscientiousness	–0.66	0.51	–0.04
Extraversion	–1.97	0.05	–0.13
Agreeableness	–0.39	0.69	–0.03
Neuroticism	–1.42	0.16	–0.09

*t*, *p*, and β values for each predictor of low prevalence accuracy

**Table 6:** Complete low prevalence reaction time regression results

	*t*	*p* value	β
High prevalence performance	7.29	<0.001	0.51
*K*	1.98	0.05	0.14
Vigilance	1.85	0.07	0.13
Attentional control	1.86	0.07	–0.14
Intelligence	0.45	0.65	0.03
Conscientiousness	–1.66	0.1	–0.11
Extraversion	–2.08	0.04	–0.15
Agreeableness	0.55	0.58	0.04
Neuroticism	–1.42	0.16	–0.09

*t*, *p*, and β values for each predictor of low prevalence reaction time

## Footnotes

1 A regression was also run using accuracy data transformed to *A*’ (Zhang & Mueller, 2005). A similar pattern of results emerged; the same five factors were significant predictors. However, we report only the corrected hit analyses because *A*’ values can be unreliable at the extremes (i.e., when performance is 0 or 100%) and over 90% of our subjects had either a hit rate of 100% or a false alarm rate of 0% in at least one condition.

2 We also performed this regression leaving low prevalence search performance out of the model. We find that only vigilance, *t* = 2.15, *p* = 0.03, β = 0.18, accounted for a significant portion of the variance. All other predictors were not significant, *t* values < 1.74, *p* values > 0.08, β values < 0.15.
